# Buried Depressed-Cladding Waveguides Inscribed in Nd^3+^ and Yb^3+^ Doped CLNGG Laser Crystals by Picosecond-Laser Beam Writing

**DOI:** 10.3390/ma17081758

**Published:** 2024-04-11

**Authors:** Gabriela Croitoru, Florin Jipa, Madalin Greculeasa, Alin Broasca, Flavius Voicu, Lucian Gheorghe, Nicolaie Pavel

**Affiliations:** 1 Laboratory of Solid-State Quantum Electronics, National Institute for Laser, Plasma and Radiation Physics, 077125 Magurele, Ilfov, Romania; madalin.greculeasa@inflpr.ro (M.G.); alin.broasca@inflpr.ro (A.B.); flavius.voicu@inflpr.ro (F.V.); lucian.gheorghe@inflpr.ro (L.G.); nicolaie.pavel@inflpr.ro (N.P.); 2 Photonic Investigations Laboratory—PhIL, Center for Advanced Laser Technology, National Institute for Laser, Plasma and Radiation Physics, 077125 Magurele, Ilfov, Romania; florin.jipa@inflpr.ro

**Keywords:** waveguide lasers, picosecond-laser beam writing, CLNGG disordered crystals, optical pumping, laser emission

## Abstract

Buried depressed-cladding waveguides were fabricated in 0.7-at.% Nd:Ca_3_Li_0.275_Nb_1.775_Ga_2.95_O_12_ (Nd:CLNGG) and 7.28-at.% Yb:CLNGG disordered laser crystals grown by Czochralski method. Circular waveguides with 100 μm diameters were inscribed in both crystals with picosecond (ps) laser pulses at 532 nm of 0.15 μJ energy at 500 kHz repetition rate. A line-by-line writing technique at 1 mm/s scanning speed was used. Laser emission at 1.06 μm (with 0.35 mJ pulse energy) and at 1.03 μm (with 0.16 mJ pulse energy) was obtained from the waveguide inscribed in Nd:CLNGG and Yb:CLNGG, respectively, employing quasi-continuous wave pumping with fiber-coupled diode lasers. The waveguide realized in RE^3+^-doped CLNGG crystals using ps-laser pulses at high repetition rates could provide Q-switched or mode-locked miniaturized lasers for a large number of photonic applications.

## 1. Introduction

The use of picosecond (ps) laser systems in ultrafast micromachining is becoming more and more attractive due to factors such as reduced costs, system stability, increased laser pulse energies, and high repetition rates. However, few works in the open literature describe the realization of waveguides in glasses and crystalline materials with laser systems providing pulses of few ps-pulse duration [1]. Zhang et al. [2] reported for the first time the fabrication with ps-laser pulses of waveguides in glasses, opening more reliable ps lasers as an alternative practical direction of femtosecond (fs) lasers. McMillen et al. [3] investigated the dependence between the waveguide’s optical quality and the duration of the pulses used for writing and concluded that for inscribing highly symmetric waveguides in lanthanum gallium sulfide chalcogenide glasses, pulses with 1.5 ps duration are more appropriate. Grivas et al. [4] reported the fabrication and laser emission from fs- and ps-laser-inscribed channel waveguides in Ti:sapphire, and Matthäus et al. [5] realized buried waveguides in single crystalline silicon by using laser pulses with duration ranging from 0.8 to 10 ps.

The crystals belonging to the large family of garnet-type crystals are excellent hosts for doping with laser-active rare-earth ions, having high thermal conductivity compared to rare-earth-doped glasses and strong absorption lines compatible with the use of laser diodes as pumping sources. Furthermore, they provide large stimulated emission cross-sections for the optically active dopant ions. Some of the garnet-type crystals that have particularly attracted attention are the structurally disordered calcium niobium gallium garnet (CNGG) and calcium lithium niobium gallium garnet (CLNGG), in which the partially disordered structure is due to the mixed occupation of some of the cationic sites with different ions as a result of their multi-component content [6,7,8]. Therefore, their natural intrinsic disorder leads to an inhomogeneous broadening of the absorption and emission lines of the doped active ions [9], while maintaining good thermo-mechanical properties comparable to those of ordered crystals such as YAG or YVO_4_. Compared to CNGG, the absence of the cationic vacancies is the main advantage of the CLNGG crystal, which increases the damage threshold. Previous studies have also shown that disordered garnets possess excellent properties for generation of nanosecond and sub-ps laser pulses, using a passive Q-switch or mode-locking technique [10,11].

The family of disordered gallium garnet crystals has different nonlinear properties compared to YAG crystals, namely the value of the nonlinear refractive index n_2_ corresponding to gallium garnets (n_2_~1.2 × 10^−19^ m^2^/W) is larger than that of YAG (n_2_~0.62 × 10^−19^ m^2^/W) [12]. When discussing light–matter interaction, the material’s nonlinear properties have a great impact on the propagation of ultrashort laser pulses because self-focusing comes into play. These nonlinearities have influence on the laser-irradiated region producing features with elongated, elliptical shapes. McMillen et al. [3] found that the use of ps pulses, rather than sub-ps pulse duration, alleviates these nonlinear effects, such as self-focusing for waveguide writing in a chalcogenide glass, a material with high nonlinearity, realizing waveguides with optimal waveguide performance. In this context, we have employed the use of ps-laser pulses for waveguide fabrication, targeting a well-defined and uniform modification in both crystals for an improved control in the waveguide shape design.

Here we present, for the first time to our knowledge, the fabrication of buried depressed-cladding waveguides in Nd-doped CLNGG (Nd:CLNGG) and Yb-doped CLNGG (Yb:CLNGG) employing laser pulses at 532 nm with 5 ps duration, delivered by a frequency-doubled Nd:YVO_4_ laser at high-repetition rates. Lasing at ~1 μm was generated when the pump was realized with fiber-coupled laser diodes at 807 nm for Nd:CLNGG and at 973 nm for Yb:CLNGG crystal. The usage of a laser source that delivers high-repetition rate ps-pulses has enabled the possibility to increase the writing speed to 1 mm/s thus, reducing the processing time and, more importantly, inscribing waveguides with reasonable levels of propagation losses. Such waveguides are good candidates for generation of ultra-short laser pulses from compact laser sources at 1 μm.

## 2. Materials and Methods

### 2.1. Crystal Growth

Active photonic devices, specifically waveguide lasers activated with rare earth ions from the lanthanide series, such as neodymium (Nd^3+^), praseodymium (Pr^3+^), holmium (Ho^3+^), or ytterbium (Yb^3+^), have been developed in the last years [13,14,15,16]. Among them, Nd^3+^ has significant importance for laser emission at 1 μm from waveguide lasers offering high optical gain and low lasing threshold. Yb^3+^ is competing with Nd^3+^ for laser emission in the 1 μm spectral region because it has a smaller quantum defect, thus the thermal load on the active medium is low and potentially allows for high-power laser generation with high slope efficiencies, broader emission bandwidth, and longer upper-laser level lifetime, enabling tunable laser emission or generation of ultrashort pulses.

In our case, the Nd:CLNGG and Yb:CLNGG crystals with Nd^3+^ and Yb^3+^ dopant ions concentrations of 0.7 at.% and 7.28 at.%, respectively, further denoted as 0.7-at.% Nd:CLNGG and 7.28-at.% Yb:CLNGG, were grown by the Czochralski method. The conventional solid-state reaction method was used to synthesize the corresponding polycrystalline Nd:CLNGG and Yb:CLNGG starting materials. CaCO_3_ (99.95% purity), Li_2_CO_3_ (99.998 purity), Nb_2_O_5_ (99.9985% purity), Ga_2_O_3_, Nd_2_O_3_, and Yb_2_O_3_ (of 5N purity) were used as raw materials. Considering that Nd^3+^ and Yb^3+^ dopant ions replace Ca^2+^ ions in the CLNGG host matrix and the values of the effective segregation coefficients (k_eff_) of Nd^3+^ and Yb^3+^ in the CLNGG crystal are 0.55 [7] and 0.91 [17], respectively, the chemical compositions of the starting materials were established to be 1.28-at.% Nd:CLNGG and 8-at.% Yb:CLNGG, respectively. The raw materials were weighed according to the chemical equations:

3CaCO_3_ + 0.13926Li_2_CO_3_ + 0.88926Nb_2_O_5_ + 1.50348Ga_2_O_3_ + 0.0192Nd_2_O_3_ → Ca_3_Li_0.275_Nb_1.775_Ga_2.95_O_12_·0.0128Nd_3_ Li_0.275_Nb_0.275_Ga_4.45_O_12_ + 3.13926CO_2_ ↑ (1)

3CaCO_3_ + 0.1485Li_2_CO_3_ + 0.8985Nb_2_O_5_ + 1.653Ga_2_O_3_ + 0.12Yb_2_O_3_ → Ca_3_Li_0.275_Nb_1.775_Ga_2.95_O_12_·0.08Yb_3_ Li_0.275_Nb_0.275_Ga_4.45_O_12_ + 3.1485CO_2_ ↑ (2)

The materials were mixed by grounding in an agate mortar, pressed into cylindrical tablets, and sintered at 1300 °C for 24 h. The growth process was carried out in an air atmosphere using platinum crucibles with an internal diameter and a height of 30 mm. All crystals were grown on crystalline seeds oriented along the ⟨111⟩ direction cut from an undoped CLNGG crystal. The growth temperature was determined to be about 1450 ± 10 °C; the pulling and rotation rates were kept constant at 1 mm/h and 15 rpm, respectively, throughout the crystal growth processes. Finally, the as-grown crystals were annealed in an air atmosphere for 24 h at a temperature of 1300 °C to eliminate the residual thermal stress accumulated during growth, as well as to stabilize the trivalent oxidation state of the Nd and Yb dopant ions.

### 2.2. Experimental Setup for Laser Emission

An experimental set-up, described in our previous works [18,19], was used to characterize the emission performances of the laser crystals. For the Nd:CLNGG medium, the optical pumping was carried out with a fiber-coupled diode laser (LIMO Co., Dortmund, Germany) with emission at 807 nm (λ_p_) wavelength, while the Yb:CLNGG crystal was pumped in the highly absorbing level at λ_p_ = 973 nm with a fiber-coupled diode laser (LIMO Co., Germany). In order to mitigate the thermal effects induced in the crystal when pumping in continuous wave (CW) mode, the two diode lasers will be operated in quasi-CW conditions (1 ms pump pulse duration, repetition rate of 5 Hz). A multimode fiber with diameter ϕ = 100 μm and numerical aperture NA = 0.22 was used to transfer the diode laser radiation in each laser medium, by imaging the beam with a focusing line containing two lenses, one of 50 mm focal length and one of 30 mm focal length.

In the setup, two plane parallel mirrors, dedicated for each crystal type, were placed very close to the lateral surfaces of the crystals and used as a linear resonator. In the case of Nd:CLNGG crystal, the resonator rear mirror was coated for high reflectivity HR (R > 0.998) at the emission wavelength (λ_em_) of 1.06 μm and with high transmission HT (T > 0.98) at λ_p_ = 807 nm. For the Yb:CLNGG crystal, we have used a rear mirror with HR coating (R > 0.998) at λ_em_ = 1.03 μm and with HT (T > 0.98) at λ_p_ = 973 nm. Out-coupling mirrors (OCM) with various transmissions T at the laser wavelength, λ_em_ were used. The gain media were consecutively positioned on a metallic plate with no further cooling. A CMOS camera was used to visualize the output surfaces of the waveguides during optical pumping. The laser beam near-field distributions were recorded with a SP620U Spiricon camera (spectral range from 190 nm to 1100 nm).

### 2.3. Waveguides Writing with Ps-Laser Pulses

**Figure 1:**
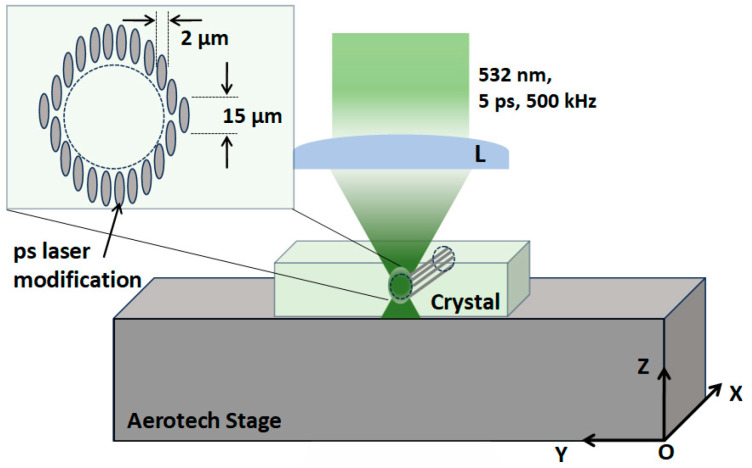
The experimental set-up used for waveguide writing in the 0.7-at.% Nd:CLNGG and 7.28-at.% Yb:CLNGG ceramic is presented. L: lens.

The experimental set-up used for waveguides writing in the Nd:CLNGG and Yb:CLNGG crystals is represented schematically in Figure 1. The beam from a ps-laser system (from Lumera, Coherent, USA), delivering pulses at 532 nm wavelength with 5 ps duration and 100 μJ maximum energy at 500 kHz repetition rate, was focused 500 μm beneath the surface of the two media with an aspheric lens (L) of 6.24 mm focal length and NA = 0.40. The focal spot (measured in air) had a diameter of ~2.0 μm. The focusing position, on the surface and inside the media, was visualized and controlled with a CCD camera. The writing process was managed by placing the two media on an Aerotech PlanarDL motorized translation stage with computer control and ±500 nm resolution. The location of the beam on the Oz axis was adjusted by controlling the focusing lens position with an additional translation stage. Extensive preliminary tests were performed over a wide range of values for laser pulse energy, motorized scanning speed and tracks separation in order to obtain the optimal laser processing parameters. Based on these results, the waveguides were inscribed with pulse energy of 0.15 μJ at 1 mm/s scanning speed. The distance between the two consecutive tracks was set at 5 μm.

## 3. Results

### 3.1. Characterization of Bulk Crystals

**Figure 2:**
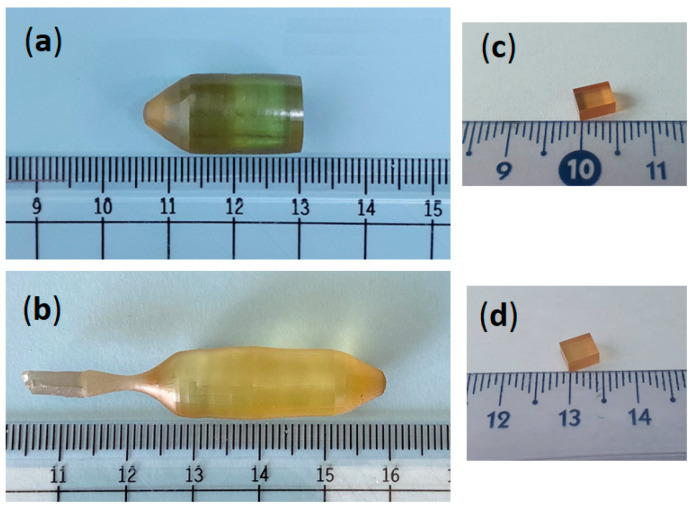
The crystal boules after growing and annealing treatment: (**a**) 0.7-at.% Nd:CLNGG; (**b**) 7.28-at.% Yb:CLNGG. (**c**) Nd:CLNGG sample with 5.9-mm thickness; (**d**) Yb:CLNGG sample with 5.0-mm thickness.

Each laser crystal was investigated by optical microscopy and prepared for further investigations, targeting the emission performances measurements and laser waveguides inscribing. A photo of the Nd:CLNGG crystal is shown in Figure 2a, while the as-grown Yb:CLNGG crystal is presented in Figure 2b. The crystals have no visible defects and no scattering centers under illumination with a He-Ne laser were observed. Several pieces, having 3.0 × 3.9 × 5.9 mm^3^ for Nd:CLNGG and 3.0 × 4.0 × 5.0 mm^3^ for Yb:CLNGG were cut from the grown crystals and polished to laser-grade quality for optical properties characterization and laser processing, as shown in Figure 2c,d, respectively.

**Figure 3:**
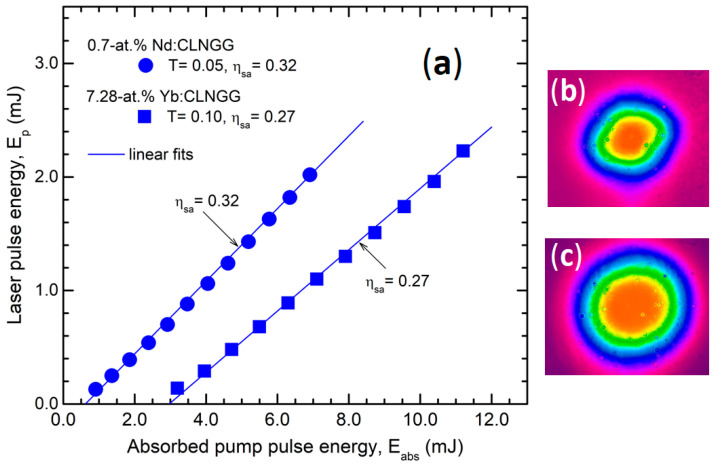
(**a**) The laser pulse energy, E_p_ versus absorbed pump pulse energy, E_abs_, for bulk Nd:CLNGG (OCM with T = 0.05 at λ_em_ = 1.06 μm) and bulk Yb:CLNGG (OCM with T = 0.10 at λ_em_ = 1.03 μm). The laser beam profile is shown for laser emission at maximum pump level in (**b**) Nd:CLNGG and (**c**) Yb:CLNGG.

Using the setup described above, we investigated the laser emission performances for bulk 0.7-at.% Nd:CLNGG at λ_em_ = 1.06 μm and for bulk 7.28-at.% Yb:CLNGG at λ_em_ = 1.03 μm, for quasi-CW pumping regime (Figure 3a). The near-field beam profiles are shown in Figure 3b for Nd:CLNGG and Figure 3c for Yb:CLNGG. For each laser crystal, the emission characteristics were determined, and the results are specified in Table 1.

### 3.2. Waveguide Fabrication by Ps-Laser Irradiation

**Figure 4:**
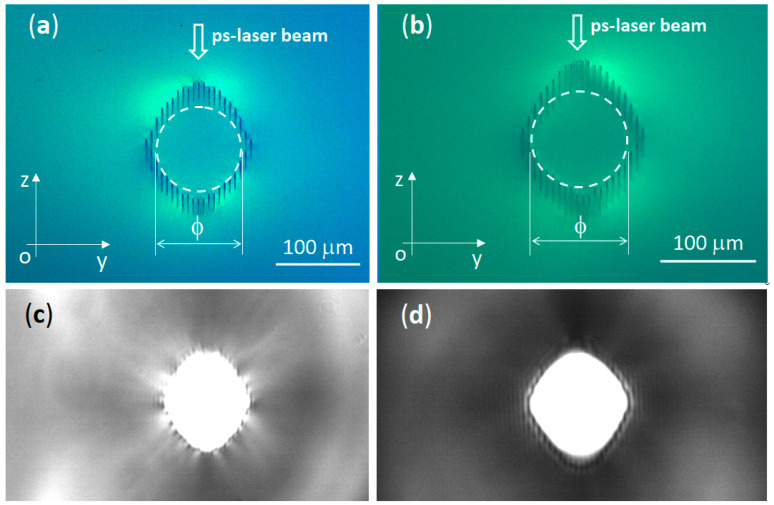
Cross-section view for (**a**) waveguide NCLNGG-C and (**b**) waveguide YCLNGG-C; each waveguide boundary is marked by white dashed line. The exit side under optical pumping is presented for (**c**) waveguide NCLNGG-C and (**d**) waveguide YCLNGG-C.

Buried depressed-cladding waveguide with circular shape and diameter ϕ = 100 μm were inscribed in Nd:CLNGG (denoted by NCLNGG-C) and Yb:CLNGG (denoted by YCLNGG-C) using the experimental setup described in Section 2.3. By inscribing parallel tracks on the Ox direction, with 1 mm/s scan speed, we have realized circular-shaped waveguides centered at 500 μm under the crystal’s surface. The 532 nm inscribing beam is translated on the crystal’s full length and the position along axis Oz is calculated and adjusted at each layer to generate a circular contour in the center. A microscope was used to record cross-section images of the waveguides, as shown in Figure 4a for NCLNGG-C and in Figure 4b for YCLNGG-C. After a qualitative evaluation, it was concluded that the tracks are smooth, and no cracks were observed. Tracks with similar sizes were inscribed in both crystals, using the same level of pulse energy of 0.15 μJ, indicating that the refractive index modification depends on the gain medium properties, on the laser exposure conditions and not on the rare-earth ion dopant. Images of the waveguide exit surfaces are illustrated in Figure 4c,d during the optical pumping; a low leakage is observed between the written tracks. Although the high repetition rate assures a reduced laser power fluctuation during the waveguide fabrication, controlling the track position overlap without inducing crystal damage remains a challenge. Nevertheless, we optimized the waveguide fabrication process by reducing the total number of tracks to 40, finishing the writing process in less than 6 min, a reduced value compared with other methods [18].

The level of propagation losses was determined for each waveguide. To do this, the beam from a HeNe laser (emission at 632.8 nm) was coupled (coupling efficiency close to unity) into each structure and the power of the transmitted light was measured. After subtracting the coupling and the Fresnel losses, the propagation losses were determined to be 0.9 dB/cm for the NCLNGG-C waveguide and 2.5 dB/cm for the YCLNGG-C waveguide, whereas for the bulk materials the corresponding losses were 0.4 dB/cm for Nd:CLNGG and 0.8 dB/cm for Yb:CLNGG. This loss level is below that obtained for buried depressed-cladding waveguides inscribed in the same crystals [20]: 2.6 dB/cm at 632.8 nm for an elliptical waveguide realized in 0.7-at.% Nd:CLNGG (5.9-mm length) and 5.6 dB/cm, for a waveguide with elliptical shape realized in a 4.3-at.% Yb:CLNGG crystal (length of 3.0 mm). The later waveguides were realized using an amplified laser system delivering pulses at 775 nm with 200 fs duration at 2 kHz repetition rate. We assume that the use of ps-laser pulses for waveguide fabrication in crystals with stronger nonlinear properties leads to the creation of a smaller number of lattice defects compared to waveguides realized with typical fs-laser systems leading to a lower level of propagation losses. We can observe that the loss level for Yb:CLNGG is higher and this may be due to the lower optical quality of this crystal. The interplay between laser pulse wavelength used for writing, pulse duration, and pulse repetition rate can contribute to the improvement of the waveguides optical properties. Also, we expect to further decrease the propagation losses by setting a lower value than 5 μm between the parallel tracks.

### 3.3. Laser Emission from Circular Waveguides

**Figure 5:**
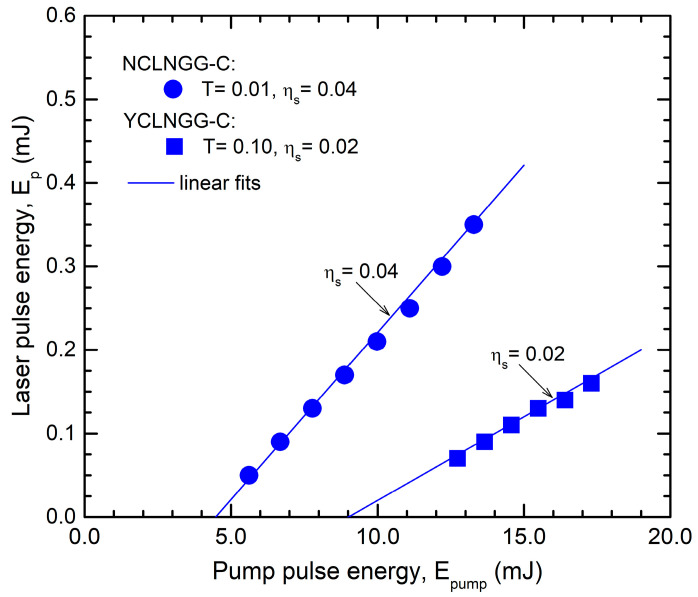
The laser pulse energy, E_p_ versus pump pulse energy, E_pump_, for NCLNGG-C (OCM with T = 0.01 at λ_em_ = 1.06 μm) and YCLNGG-C (OCM with T = 0.10 at λ_em_ = 1.03 μm).

Laser emission was generated for the waveguides inscribed in the two gain media. A high coupling efficiency (close to one) of each pump beam into the corresponding waveguide was achieved by carefully adjusting the pump-beam focusing position inside each laser crystal. A maximum laser pulse energy of E_p_ = 0.35 mJ was determined from the NCLNGG-C waveguide for E_pump_ = 13.3 mJ (the overall optical-to-optical efficiency was η_o_~0.03); the laser operated with a slope efficiency η_s_ = 0.04 (Figure 5). The YCLNGG-C waveguide yielded laser pulses with maximum energy E_p_ = 0.16 mJ for E_pump_ = 17.3 mJ (i.e., η_o_~0.01) and η_s_ = 0.02. The laser beam profiles corresponding to each waveguide were highly multimode.

## 4. Discussion

In this report, we describe for the first time the usage of high repetition rate ps-laser for waveguide fabrication starting with the crystal grown and ending with waveguide characterization. The increased interest in such devices is largely due to the compact geometry of the waveguide structures, allowing for light confinement and propagation in an extremely small volume of micrometric (μm) scale and, in this way, achieving intensity levels considerably higher than those in bulk materials. By combining the properties of waveguide structures with those of active laser media, miniaturized laser sources of increased potential for integrated optics can be realized with important advantages in terms of high optical gain and implicitly low lasing threshold, better management of thermal effects, and, thus, compatibility with laser diodes for operation at high power levels or beam shaping.

Waveguides were inscribed in various crystalline media with both types of morphological changes: Type I for positive refractive index modification (Δn > 0) and Type II for negative refractive index modification (Δn < 0). Type I modification is specific to glasses and several crystals, like LiNbO_3_ [21], KTP [22], Nd:YCOB [23], BGO [24], LiTaO_3_ [25], or Nd:YAG [26]. With Type II refractive index change, waveguides are realized in crystalline as well as in ceramic media with different geometries, such as depressed-cladding waveguides in Nd:YAG [27], two-line configuration in Yb:YAG [28] or optical-lattice-like layout in Nd:YAP [29]. This approach is suitable for rare-earth doped crystals because the influence of the laser–matter interaction is minimal in the waveguide core, preserving the spectroscopic properties. Moreover, buried depressed-cladding waveguides are suited for optical pumping with fiber-coupled diode lasers with emission in the infrared region and for further power scaling. Regarding waveguide realization in disordered gallium garnet crystals, there are a couple of reports from Tan et al. on laser emission at different wavelengths from waveguides inscribed in Nd-doped CNGG crystal [30,31].

The use of ps-pulsed laser installations to realize waveguide structures generates 1.06 μm laser emission with output pulse energy E_p_ = 0.35 mJ and slope efficiency η_s_ = 0.04 for the circular waveguide with ϕ = 100 µm diameter realized in Nd:CLNGG and laser emission at the wavelength of 1.03 μm with output pulse energy E_p_ = 0.16 mJ and slope efficiency η_s_ = 0.02 for Yb:CLNGG. The value for η_s_ is comparable with the work conducted by Tan et al. [30]. The propagation losses at 632.8 nm were evaluated to be 0.9 dB/cm for the waveguide realized in the Nd:CLNGG and 2.5 dB/cm for the one inscribed in the Yb:CLNGG crystal. Table 2 summarizes the conditions used to inscribe buried depressed-cladding waveguides with large diameter in different active media, using fs- and ps-laser pulses. The propagation losses are also indicated, these being measured by coupling in the waveguides a HeNe laser beam at 632.8 nm. It can be seen that, in general, the losses are similar, but writing with ps-laser pulses was performed at a higher repetition rate than that with fs-laser pulses, which ensured a faster inscribing speed.

Although we have used the same laser exposure conditions, the losses between the two crystals may be due to factors such as the crystal’s lower optical quality or even the refractive index modification realized by the ps laser. On the other hand, propagation losses could be reduced by inscribing depressed-cladding waveguides with an “ear-like” structure. This configuration, recently proposed, allowed efficient laser emission in Nd:YAG [38] or Pr:YLF [39]. The physical processes initiated during crystal irradiation by the high repetition rate ps laser are still under debate, and most of the time the fs model is used, which is based on nonlinear photoionization processes. In this case, the excited carriers will linearly absorb photons and subsequently impact ionized bound electrons, thus generating an electron avalanche. Then, the accumulated energy is transferred to the irradiated material through electron-photon scattering on a ps time scale and carried out via thermal diffusion, finally inducing moderately strong refractive index modifications in the range of Δn = 10^−4^ to 10^−2^ [40,41]. For sub-ps laser pulses, the nonlinear absorption phenomena take place on a shorter scale than the electron–phonon scattering time and, in this way, heat diffusion outside the focal area is minimized.

For longer pulse duration, as is the situation with pulses ranging from 1 to 10 ps, the nonlinear photo-ionization processes occur on the same time scale as the energy transfer process to the lattice via phonon scattering; consequently, a heat accumulation effect appears. This effect may be deleterious regarding the optical properties of the waveguide structures. However, the interplay of other laser parameters such as laser pulse energy, repetition rate, and scan speed, whose values show significant importance in the optimization process of these properties, has the capability to reduce the negative impact of longer pulse durations. This interplay of laser parameters allows the usage of both fs- and ps-laser systems delivering pulses at low to moderate (1–200 kHz) and, also, at high repetition rates (>200 kHz). At high repetition rates, heat builds up around the irradiated volume because the time between the laser pulses is shorter than the time for the accumulated energy to diffuse out of the volume. It has been reported that waveguides written in this regime have superior optical properties (in terms of mode symmetry and lower propagation losses) than those inscribed in the low repetition rate regime due to the presence of a rapid annealing effect. These improved results have been successfully explained in terms of a partial recombination of defects for waveguides realized in Yb:YAG ceramic laser gain medium [42].

## 5. Conclusions

Buried depressed-cladding waveguides with circular geometry have been realized in 0.7-at.% Nd:CLNGG and 7.28-at.% Yb:CLNGG disordered crystals by ps-laser beam exposure, for the first time to our best knowledge. The laser emission performances at 1.06 μm and 1.03 μm were evaluated under the pump at 807 nm (for Nd:CLNGG) and at 973 nm (for Yb:CLNGG) with a fiber-coupled diode lasers. Circular cladding waveguides of 100 μm diameter realized in Nd:CLNGG and Yb:CLNGG crystals delivered laser pulses at 1.06 μm with 0.35 mJ output energy, respectively, at 1.03 μm with 0.16 mJ output energy. The slope efficiencies were 0.04 and 0.02 for the two waveguides, respectively. The waveguide realized in RE^3+^-doped CLNGG crystals using ps-laser pulses at high repetition rates could provide miniaturized pulsed laser sources (Q-switched or mode-locked) by further integrating a suitable saturable absorber. The direct laser writing technique was used to inscribe waveguides in these crystals and proved once again its ability to induce refractive index modifications in a multitude of dielectric materials, even if the laser – matter interaction is a rather complex process and the phenomena as well as the structural effects are not completely understood. The growth by the Czochralski technique of Nd- and Yb-doped CLNGG disordered laser crystals with superior optical quality is among our current research priorities. These new crystals will allow inscribing buried depressed-cladding waveguides with different sizes, as well as scaling of laser emission using the pump with fiber-coupled diode lasers.

## Figures and Tables

**Table 1:** Characteristics of laser emission at 1.06 μm from bulk 0.7-at.% Nd:CLNGG (OCM with T = 0.05) and at 1.03 μm from bulk 7.28-at.% Yb:CLNGG (OCM with T = 0.10).

Laser Crystal	Absorbed Pump Pulse Energy, E_abs_ (mJ)	Absorption Efficiency, η_a_	Laser Pulse Energy, E_p_ (mJ)	Optical Efficiency, η_oa_	Slope Efficiency, η_sa_
0.7-at.% Nd:CLNGG	6.90	0.52	2.0	0.29	0.32
7.28-at.% Yb:CLNGG	11.20	0.88	2.20	0.27	0.27

**Table 2:** A comparison of propagation losses for buried depressed-cladding waveguides with large diameter that were inscribed by linear translation technique [27] in different laser media is presented. The losses were determined at 632.8 nm with a HeNe laser. λ: laser beam wavelength; t_p_: laser pulse duration; E_p_: pulse energy; f: repetition rate.

Laser Crystal	Waveguide Diameter, ϕ (μm)	Propagation Loss (dB/cm)		Characteristics of the Writing Beam					Ref.
		Bulk	Waveguide	λ (nm)	t_p_	E_p_ (μJ)	f (kHz)	Writing Speed	
Nd:YAG crystal	elliptical 116 μm × 110 μm	-	0.12	800	110 fs	1.2–1.5	1.0	0.5–0.7 mm/s	[32] *
Nd:YAG ceramic	~100	-	0.8	800	120 fs	0.2–0.4	1.0	700 μm/s	[33]
Nd:GGG crystal	90 120 150	-	2.5 2.0 1.7	795	120 fs	2.1	1.0	500 μm/s	[34]
Nd:GdVO_4_	150		0.7	795	120 fs	1.68	1.0	500 μm/s	[35]
Nd:YVO_4_	100	-	1.5–2.4	775	200 fs	0.3	1.0	50 μm/s	[36]
Nd:YAG ceramic	50 100	0.2	1.0–1.2 1.5–1.8	775	200 fs	1	2.0	50 μm/s	[18]
Nd:YAG ceramic	100	0.3	0.9	532	5 ps	0.2	500	1 mm/s	[37]
Nd:CLNGG Yb:CLNGG	100	0.4 0.8	0.9 2.5	532	5 ps	0.15	500	1 mm/s	this work

* In this work, the propagation loss was determined from the threshold of laser emission.

## Data Availability

Data underlying the results presented in this paper may be obtained from the authors upon reasonable request.
